# Continental‐wide prioritization of protected areas to address global and habitat‐specific goals for avian biodiversity conservation

**DOI:** 10.1111/cobi.70324

**Published:** 2026-05-14

**Authors:** Yanjie Xu, Diego Pavón‐Jordán, Tyler Hallman, Tristan R. M. Bakx, Lluís Brotons, Sergi Herrando, Alison Johnston, Brett K. Sandercock, Thomas Sattler, Alaaeldin Soultan, Vitalie Ajder, Dawn E. Balmer, Brian Burke, Thiago Cavalcante, Tomasz Chodkiewicz, Ruud P. B. Foppen, Anthony D. Fox, Verena Keller, Tatiana Kornienko, Peter Knaus, Heini Kujala, Åke Lindström, Qenan Maxhuni, Károly Nagy, Ingar Jostein Øien, Tomas Pärt, Dimitrije Radišić, Draženko Z. Rajković, Iván Ramirez, Paul Shimmings, Karel Šťastný, Thomas Vikstrøm, Tomasz Wilk, Aleksi Lehikoinen

**Affiliations:** ^1^ Finnish Museum of Natural History University of Helsinki Helsinki Finland; ^2^ Finnish Environment Institute (Syke) Helsinki Finland; ^3^ Division of Land and Biodiversity Norwegian Institute for Nature Research (NINA) Trondheim Norway; ^4^ School of Environmental and Natural Sciences Bangor University Bangor UK; ^5^ CREAF Cerdanyola del Vallès Spain; ^6^ CSIC Cerdanyola del Vallès Spain; ^7^ CTFC Solsona Spain; ^8^ European Bird Census Council Prague Czech Republic; ^9^ Catalan Ornithological Institute Natural Sciences Museum of Barcelona Barcelona Spain; ^10^ Centre for Research into Ecological and Environmental Modelling, School of Mathematics and Statistics University of St Andrews St Andrews UK; ^11^ Swiss Ornithological Institute Sempach Switzerland; ^12^ Institute of Ecology and Geography Moldova State University Chisinau Republic of Moldova; ^13^ “Al. I. Cuza” Univ., ICI RECENT AIR Center, Lab. of Interdisciplinary Research on the Marine Environment and Marine‑Terrestrial Atmosphere “Prof. Dr. Ioan Borcea” Marine Biological Station Constanța Romania; ^14^ British Trust for Ornithology Thetford UK; ^15^ BirdWatch Ireland, Bullford Business Campus Kilcoole, Co. Wicklow Republic of Ireland; ^16^ Polish Society for the Protection of Birds Marki Poland; ^17^ Museum and Institute of Zoology Polish Academy of Science Warsaw Poland; ^18^ Sovon Dutch Centre for Field Ornithology Nijmegen The Netherlands; ^19^ Department of Ecology, RIBES Radboud University Nijmegen The Netherlands; ^20^ Department of Ecoscience Aarhus University Aarhus Denmark; ^21^ Department of Biology Lund University Lund Sweden; ^22^ Kosovo Ornithological Society Prishtina Republic of Kosovo; ^23^ MME BirdLife Hungary Budapest Hungary; ^24^ BirdLife Norway Trondheim Norway; ^25^ Department of Ecology The Swedish University of Agricultural Sciences Uppsala Sweden; ^26^ Faculty of Science, Department of Biology and Ecology University of Novi Sad Novi Sad Serbia; ^27^ National Institute of the Republic of Serbia, Department of Biodiversity and Environmental Research University of Belgrade – Institute for Multidisciplinary Research Belgrade Serbia; ^28^ Secretariat of the Convention on Migratory Species Bonn Germany; ^29^ Faculty of Environmental Sciences Czech University of Life Sciences Prague Czech Republic; ^30^ BirdLife Denmark Copenhagen Denmark

**Keywords:** animals, area‐based conservation, biodiversity target, birds, distribution, Europe, prioritization, protected areas, Animales, áreas protegidas, aves, conservación por zonas, distribución, establecimiento de prioridades, Europa, objetivo de biodiversidad

## Abstract

The United Nations and European Union have set ambitious conservation goals to halt and reverse declines in biodiversity by protecting 30% of land and sea areas by 2030. Effective conservation planning requires evidence‐based spatial prioritization to maximize the coverage of species within designated protected areas. Based on a pan‐European database for occurrence and abundance of breeding birds collected in the 2010s, we applied the Zonation algorithm to identify key areas that would maximize the protection of ranges and populations for 435 species of breeding birds across Europe using either continental or national prioritization targets. When 30% of Europe's highest priority terrestrial areas were selected by the algorithm, 49% of species’ ranges and 63% of species’ populations were protected. When 10% and 30% of the highest priority lands were selected, compared with prioritization using occurrence data, prioritization using abundance data resulted in a higher percentage of species’ populations, especially rare and range‐restricted species, being represented for protected area coverage. Stratifying prioritization by habitat criteria greatly enhanced habitat‐specific conservation efficiency, enabling coverage of over 80% of breeding bird species’ populations in tundra, Mediterranean, and coastal habitats with a selection of 10% of the highest priority areas for each. Our prioritization supports international targets adopted under the Kunming–Montreal Global Biodiversity Framework by identifying key areas and providing a roadmap to guide optimal site protection and conservation planning in Europe.

## Abstract (es)

Establecimiento de prioridades a escala continental en materia de áreas protegidas para alcanzar los objetivos globales y específicos de cada hábitat en la conservación de la biodiversidad aviar

**Resumen:**

Las Naciones Unidas y la Unión Europea se han fijado ambiciosos objetivos de conservación para detener y revertir la pérdida de biodiversidad mediante la protección del 30 % de las zonas terrestres y marinas para 2030. Una planificación eficaz de la conservación requiere una priorización espacial basada en datos empíricos para maximizar la cobertura de las especies dentro de las áreas protegidas designadas. A partir de una base de datos paneuropea sobre la presencia y abundancia de aves reproductoras recopilada en la década de 2010, aplicamos el algoritmo Zonation para identificar áreas clave que maximizaran la protección de las áreas de distribución y las poblaciones de 435 especies de aves reproductoras en toda Europa, utilizando objetivos de priorización tanto a escala continental como nacional. Cuando el algoritmo seleccionó el 30 % de las áreas terrestres de mayor prioridad de Europa, se protegió el 49 % de las áreas de distribución de las especies y el 63 % de sus poblaciones. Al seleccionar el 10 % y el 30 % de las tierras de mayor prioridad, en comparación con la priorización basada en datos de presencia, la priorización basada en datos de abundancia dio lugar a un mayor porcentaje de poblaciones de especies —especialmente especies raras y de área de distribución restringida— representadas en la cobertura de las áreas protegidas. La estratificación de la priorización según criterios de hábitat mejoró considerablemente la eficiencia de la conservación específica de cada hábitat, permitiendo cubrir más del 80 % de las poblaciones de especies de aves reproductoras en los hábitats de la tundra, el Mediterráneo y las costas con una selección del 10 % de las áreas de mayor prioridad para cada uno. Nuestra priorización respalda los objetivos internacionales adoptados en el Marco Global de Biodiversidad de Kunming‐Montreal al identificar áreas clave y proporcionar una hoja de ruta para orientar la protección óptima de los sitios y la planificación de la conservación en Europa.

## INTRODUCTION

Global biodiversity, threatened by diverse factors such as habitat loss and degradation, climate change, overexploitation, pollution, and invasive species, is in crisis (Harfoot et al., 2021). Responding to these escalating pressures, the Convention on Biological Diversity's Kunming–Montreal Global Biodiversity Framework and the EU's Biodiversity Strategy established targets to protect 30% of land and sea by 2030 (hereafter, 30×30 target) to halt and reverse biodiversity loss (Convention on Biological Diversity, 2024). However, resources available for nature conservation are scarce at national and regional levels. Efficient, evidence‐based spatial prioritization is therefore essential for achieving the 30×30 target (Visconti et al., 2016) and provides a foundation for development of systematic conservation planning frameworks (Kukkala & Moilanen, 2013; Moilanen et al., 2022).

Complementarity‐based conservation planning tools such as Zonation and Marxan have been widely used to identify sets of sites that together maximize biodiversity representation while accounting for costs and other constraints (Giakoumi et al., 2025; Lehtomäki & Moilanen, 2013; Watts et al., 2021). At a continental scale, however, most applications have relied on species’ occurrence or expert‐drawn range maps because broadscale standardized abundance data are rarely available (Di Marco et al., 2017; Soultan et al., 2022). Occurrence‐based models can overestimate species’ distributions and fail to identify demographic strongholds that are critical for long‐term population persistence and genetic diversity (Mergeay, 2024; Wilson et al., 2006). Recent work has demonstrated that abundance‐informed models can substantially improve spatial and temporal prioritization of conservation resources (Johnston et al., 2015, 2020; Wolff et al., 2023), yet abundance‐based prioritization has rarely been implemented at a continental scale. The extent to which continent‐wide abundance data, rather than occurrence data alone, alter the spatial identification of priority areas when analyzed with complementarity‐based tools remains unknown. Furthermore, prioritization can include either national or international conservation targets (e.g., 30% land cover), and it is not well understood how these might influence continental‐scale prioritization.

As key threats vary across ecosystems, habitat‐specific conservation efforts are essential (Sutherland et al., 2009). In Europe, agricultural intensification that involves water diversions and increased use of agricultural chemicals has been identified as a major threat to native species (Rigal et al., 2023). Although many threats such as infrastructure development and urbanization transcend habitat boundaries and can be addressed through more general approaches, some threats such as agricultural intensification are habitat specific. For habitat‐specific threats, broad conservation targets alone may overlook important differences in ecosystem vulnerabilities and needs (Cobb et al., 2024). Recent continental‐scale work on European birds has shown that habitat and ecological specialization are strongly associated with population trends and conservation (Benedetti et al., 2022; Morelli et al., 2020) and that some guilds of birds in major habitat types are in steep decline (Gregory et al., 2019). Nonetheless, habitat‐specific spatial prioritization—where separate optimization is carried out for habitat‐based species groups—has not been systematically contrasted with a single‐species prioritization across a continent. Such comparisons, made possible by the availability of standardized, continental‐scale biodiversity data, are essential to assessing the potential added value of habitat‐specific approaches for large‐scale conservation planning.

Birds are central to conservation planning in Europe and globally, and major policy frameworks—including the EU Birds and Habitats Directives, Bern Convention, Agreement on the Conservation of African‐Eurasian Migratory Waterbirds, Convention on Migratory Species, and Ramsar Convention—mandate the protection of sensitive species and their habitats. Special protection areas (SPAs) under the Birds Directive and special areas of conservation (SAC) under the Habitats Directive form the Natura 2000 network, covering ∼19% of EU land area. In addition, the Emerald Network covers an additional 11–12% of the national territories of 16 non‐EU European countries (Council of Europe, 2017). Important Bird and Biodiversity Areas (IBAs), identified by BirdLife International, have frequently guided the designation of protected areas, making IBAs a critical pillar of Europe's area‐based conservation strategy (BirdLife International, 2025a). As highly mobile species capable of making rapid, fine‐grained choices between disturbance‐free and resource‐rich habitats, birds are effective indicators of changes in habitat quality (Gregory et al., 2005). Birds are also valuable as umbrella species for which conservation benefits a wide range of coexisting taxa and ecosystem processes (Roberge & Angelstam, 2004). Furthermore, the quality of occurrence and abundance data for birds is unrivalled across taxonomic groups (Keller et al., 2020), making birds a unique basis for systematic conservation planning.

In Europe, a substantial body of work has evaluated how well existing protected area networks, especially Natura 2000, Emerald, and nationally designated areas, represent vertebrate species and their habitats (Gaget et al., 2022; Hermoso et al., 2012; Kukkala et al., 2016; Princé et al., 2021). Several regional and taxon‐specific studies have used Zonation or related tools to identify bird conservation priorities and assess protected area effectiveness (Prieto‐Torres et al., 2018; Ramírez‐Albores et al., 2021), and recent pan‐European analyses have quantified patterns of avian specialization and their coverage by Natura 2000 protected areas (Benedetti et al., 2022). Despite extensive research on bird conservation and protected areas in Europe, most studies have relied on occurrence or trait data, focusing on particular regions or subsets of species, rather than on continent‐wide abundance information. A pan‐European, abundance‐informed prioritization process has not been previously conducted for the entire breeding bird community. The European Breeding Bird Atlas 2 (EBBA2) provides systematic data on the occurrence and abundance of all the breeding bird species across Europe between 2013 and 2017 (Keller et al., 2020). The 50 × 50‐km resolution of EBBA2 may not capture fine‐scale ecological processes, but it can effectively determine habitat availability and climatic suitability for highly mobile species, thereby identifying continental‐scale priority regions as a robust foundation for subsequent prioritization of important areas for biodiversity conservation at a local scale (McGill, 2010). EBBA2 therefore offers, for the first time, standardized continent‐wide abundance data for all European breeding birds, yet it has been used mainly to describe patterns of distribution, change, and specialization rather than to conduct fully abundance‐informed spatial prioritization at the European scale (Benedetti et al., 2022).

Taken together, the current knowledge gaps motivated the four specific research questions addressed in this study: How do conservation priorities for European breeding birds differ when based on abundance rather than on occurrence in a complementarity‐based optimization framework? How do conservation priorities for European breeding birds differ when 30% targets are prioritized to fill only continental‐scale targets or both national‐ and continental‐scale targets? How do separate habitat‐specific prioritizations for six major habitat groups compare with an overall full‐species prioritization in terms of spatial patterns and efficiency of population coverage? How well do existing networks of protected areas and IBAs capture the priorities identified by abundance‐based and habitat‐specific prioritizations?

We used comprehensive data from the EBBA2 program to identify key areas for bird conservation across Europe and occurrence and abundance maps for 435 species of breeding birds as inputs for the Zonation algorithm (Moilanen et al., 2022). By further identifying priority regions for six species groups with similar habitat associations such as agriculture/grassland and wetland birds, we provided a new basis for developing targeted conservation strategies that account for habitat‐specific needs. Last, we evaluated the overlap between the new priority areas and existing networks of protected areas to identify gaps where conservation efforts could be improved. Our results demonstrated how abundance‐based, habitat‐specific prioritization can yield more efficient conservation outcomes for certain species groups. We offer new insights for addressing trade‐offs between maximizing species and population coverage across all species given the limited scope for expanding protected areas where only ∼4–6% of land remains available for reaching the 30×30 target in Europe.

## METHODS

### Study area

**FIGURE 1:**
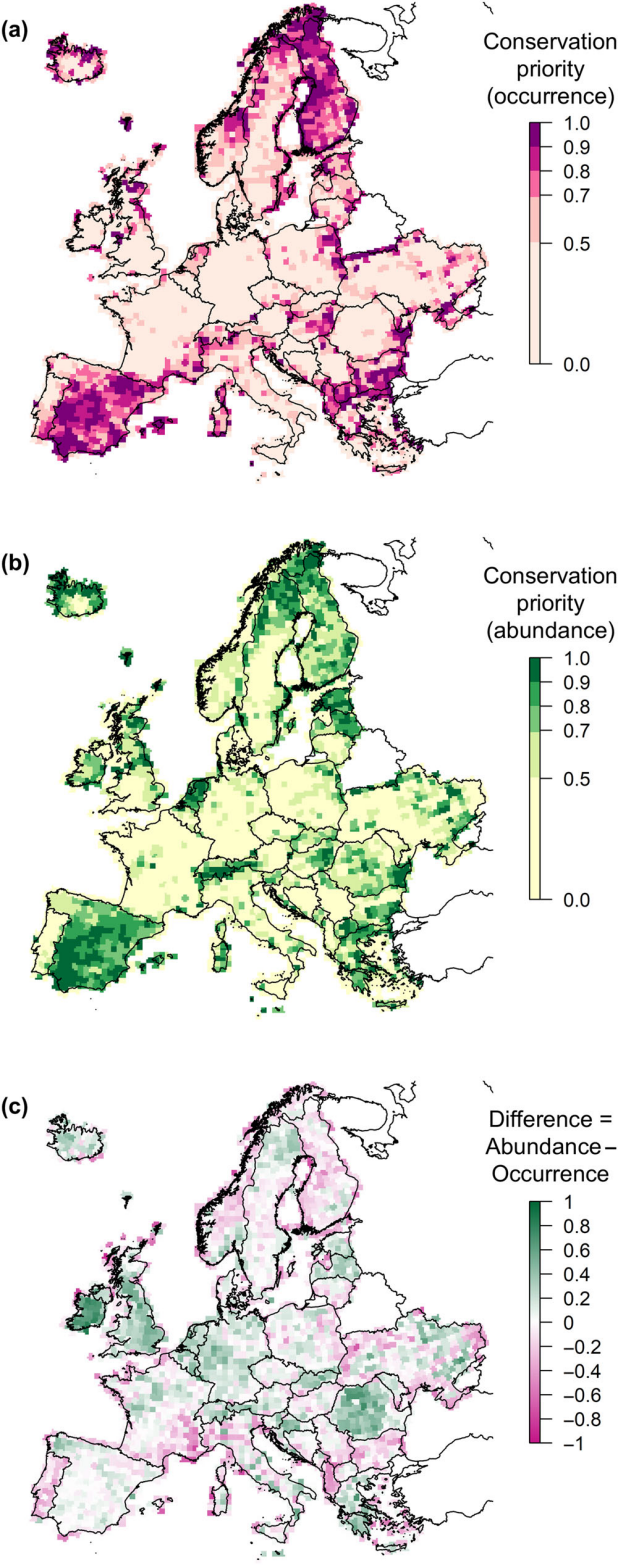
Priority areas for European breeding birds based on (a) occurrence and (b) abundance data, with 1 ranked as the highest priority and 0 as the lowest priority, and a difference map (c) showing the difference in priority ranks based on abundance versus occurrence data (cells with a positive value [green] were more important in the abundance prioritization; cells with a negative value [purple] were more important in the occurrence prioritization).

The analysis included 41 European countries with sufficient national survey efforts in EBBA2 (Figure 1). Four other countries, Belarus, Russia, Turkey, and Vatican City, contributed data to EBBA2 but were excluded because nationwide sampling effort was low. For country‐level summaries, we used 36 countries with a national area ≥2500 km^2^, which covers more than one 50 × 50‐km cell, thereby excluding Andorra, Liechtenstein, Malta, Monaco, and San Marino.

### Pan‐European breeding bird data

We used both occurrence and abundance data from the EBBA2 European breeding bird surveys as inputs to the spatial prioritization analysis. EBBA2 includes data for 625 bird species collated during the 5‐year period of 2013–2017 for 50‐km grid cells across Europe (Keller et al., 2020). Abundance within a cell was estimated to be in one of seven ordinal categories: 0; 1–9; 10–99; 100–999; 1000–9999; 10,000–99,999; and ≥100,000 pairs. We excluded cell–species combinations that lacked sufficient data on abundance estimates, and they were coded as NA. Each cell was assigned a value of 0 or 1 for species’ occurrence maps and geometric means of maximum and minimum values of each abundance interval for abundance maps.

The overall full‐species analysis excluded non‐native species and vagrant species without breeding records within the study area, which reduced occurrence and abundance maps from 625 to 435 species. ​​​Some of the remaining species were introduced to some regions but are native elsewhere in Europe. Within the introduced range, we manually assigned their occurrence and abundance to zero to prevent prioritization based on non‐native populations. Specifically, we excluded records of five introduced populations, including greater white‐fronted goose (*Anser albifrons* [Scopoli, 1769]), pink‐footed goose (*Anser brachyrhynchus* [Baillon, 1834]), and bean goose (*Anser fabalis* [Latham, 1787]) from central Europe; red‐legged partridge (*Alectoris rufa* [Linnaeus, 1758]) from the United Kingdom; and ruddy shelduck (*Tadorna ferruginea* [Pallas, 1764]) from outside of southeastern Europe. For habitat‐specific analyses, we used information on the primary habitat type of each bird species as adapted by Tucker and Evans (1997) for EBBA2 (Keller et al., 2020). The six habitat categories included agriculture and grassland (74 species); boreal and temperate forest (61); tundra, mirelands, and moorland (42); Mediterranean habitat (39); marine and coastal habitat (49); and inland wetland (62). Mountain specialists (eight species) and generalist species not predominantly associated with the six habitat categories (100) were excluded (Keller et al., 2020), leaving 327 species for habitat‐specific analysis.

### Spatial conservation prioritization

To identify priority areas for Europe's avian conservation, we used an iterative conditional sort algorithm in Zonation software to produce a complementarity‐based priority ranking of all grid cells (Moilanen et al., 2022). The algorithm starts with an initial order of the 50‐km cells and then refines the order by iteratively recalculating and adjusting marginal loss values for conservation based on species’ range and population status. The algorithm ensures that the ranking converges to an optimal solution by considering how much of each feature is present in the remaining landscape. After the iterations converge, Zonation generates priority ranks for each grid cell based on their order of importance bounded between 0 for the lowest and 1 for the highest ranks, prioritizing areas with the highest cumulative value for covering bird ranges or populations. We used the additive benefit function (ABF) as the marginal loss rule with the default exponent (0.25), which emphasizes a high average coverage across all species (Moilanen, 2007).

To retain both threatened and common species while accounting for the global significance of their European populations, each species was weighted in the prioritization by their European conservation status using four categories of the Species of European Conservation Concern (SPEC) (Burfield et al., 2023). Use of rankings gave higher weight to globally important species with a larger proportion of their global population restricted to Europe. We assigned weights based on their listed category as follows: 4 (SPEC1: species of global concern), 3 (SPEC2: European concern and concentrated in Europe), 2 (SPEC3: European concern but not concentrated in Europe), and 1 (non‐SPEC; Appendix S12).

We first performed the spatial prioritization for the complete community of all 435 species to identify important areas for avian conservation (hereafter, full prioritization). We then ran six separate prioritization analyses for the subsets of bird species associated with the six major habitats (Keller et al., 2020; Tucker & Evans, 1997) to identify important areas for conservation of each type of habitat (hereafter, habitat‐specific prioritization). We also summarized the mean priority ranks within each of the 36 included countries to assess country‐specific conservation values for birds. We repeated the spatial prioritizations separately for species’ occurrence and abundance data to evaluate and compare region‐specific conservation values for species’ ranges versus populations. Further, to identify country‐level important areas for bird conservation, we subset the priority ranks of the 50‐km cells to each country's range and selected the top 30% of highest priority areas for each country. We then compared these country‐level top 30% priority areas with the continental‐level top 30% priority areas using spatial overlap (Figure 3).

**FIGURE 2:**
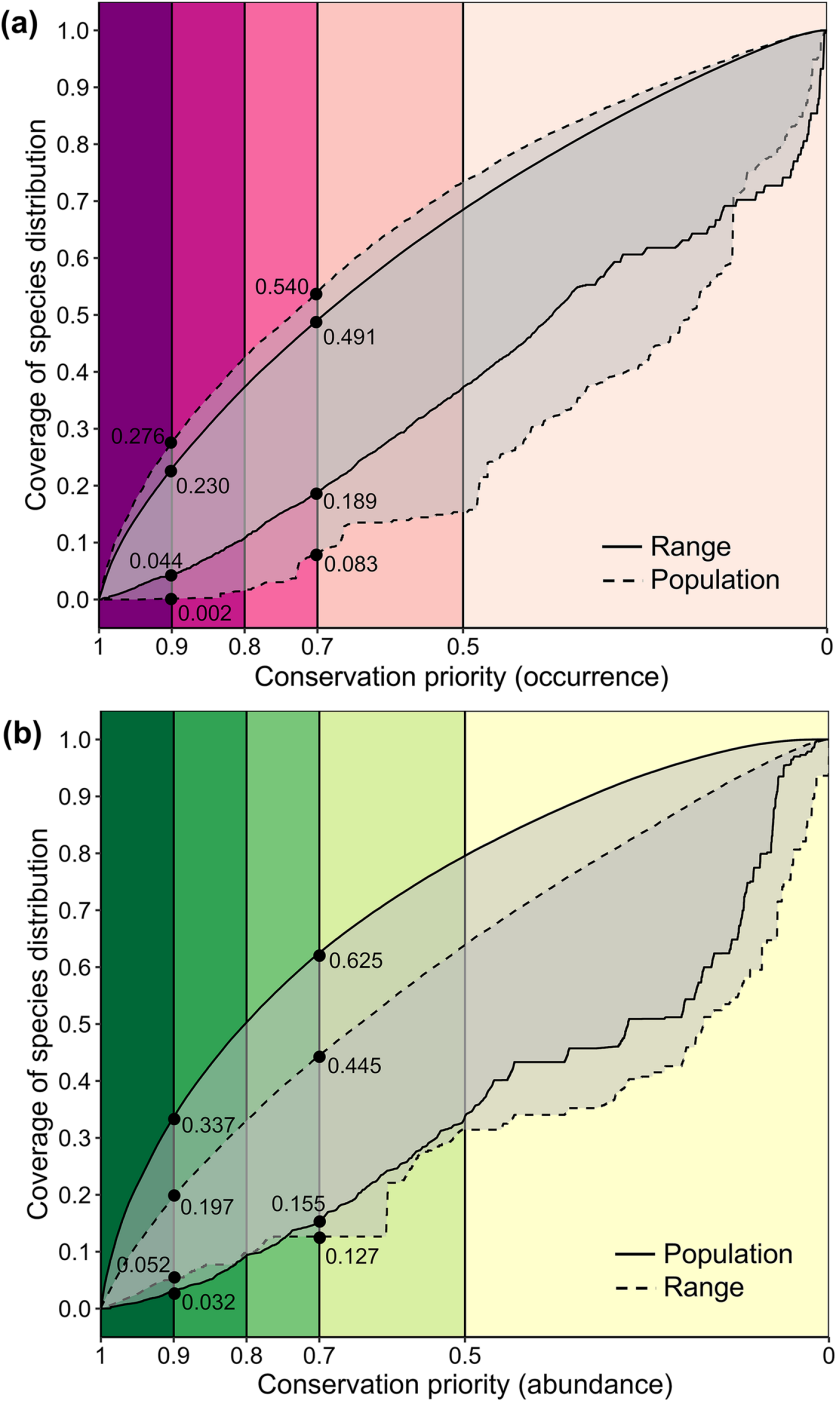
Performance curves indicating the degree of the species’ range and population covered at different conservation priority ranking levels (1–0). Lines are mean (upper lines) and minimum (lower lines) coverage for species’ ranges and populations in the most important areas when prioritization was conducted with species’ (a) occurrence and (b) abundance maps, and mean and minimum coverage were calculated among all the 435 species of breeding birds included in the full prioritizations.

To compare the overall efficacy of occurrence‐ versus abundance‐based prioritization, we calculated species’ coverage in Zonation, based on range and population, using 10% and 30% thresholds for cells with highest priority rank (Figure 2). We refer to EBBA2 data as occurrence and abundance data when used as inputs to the Zonation analyses and as range and population data when conducting the coverage assessments. The coverage for species’ range was calculated by dividing the number of cells with species’ occurrence within the priority areas by the number of cells with occurrence across the study area. The coverage for species’ population was calculated by dividing the sum of abundance levels among the top‐priority cells by the total abundance among all cells.

We conducted sensitivity analyses to test the influence of three alternative species weighting schemes (SPEC, IUCN, and unweighted) and three alternative algorithms for calculating marginal loss in Zonation (ABF and core‐area zonation [CAZ2 and CAZMAX]). To first test the sensitivity of the different weighting systems, we compared prioritization results under three scenarios: species weighted by their SPEC category, species weighted by their IUCN Red List category of extinction risk at the European scale (BirdLife International, 2021; Appendix S13), and unweighted species (details given in Appendix S3). We also compared output prioritization scores using different marginal loss rules that placed greater emphasis on range‐restricted species. ABF, CAZ2, and CAZMAX define how marginal loss from cell removal is calculated. The ABF rule aggregates losses across all species, whereas core‐area rules focus on the maximum loss from any species. The CAZ2 rule applies a balanced aggregation of maximum‐loss values to improve overall and minimum species coverage. In contrast, the CAZMAX rule adopts the strictest core‐area approach, safeguarding core habitats of the most range‐restricted species (details in Appendix S4). For each scenario, we compared resulting priority ranks using pairwise difference maps to evaluate the magnitude and spatial distribution of discrepancies.

### Analysis of Europe's protected area network

We obtained polygon shapefiles defining the extents of protected areas from Natura 2000, Emerald Network version 2022, and the Nationally Designated Areas version 2023–2024 from the Datahub of European Environment Agency (2025). Natura 2000 covers all 27 EU countries, whereas the Emerald Network covers 16 non‐EU countries, including Norway, Switzerland, Ukraine, Georgia, and other countries that are Parties to the Bern Convention but not EU members (Council of Europe, 2017). We combined the three spatial datasets to create a map of the current protected area network of Europe. We calculated the percentage area covered by the protected area network within each 50‐km cell and within each country. The protected area coverage within each country was compared with their mean priority ranks to evaluate each country's conservation efficiency. We then calculated the protected ratio by dividing the percentage of protected area within each cell or each country by the percentage of protected area within the entire study area for the 41 European countries (Figure 1).

In addition to the protected areas, we calculated the coverage of IBAs using the digital boundaries from BirdLife International (2025a). Next, we calculated the percentage area of each 50‐km cell and each country that overlapped with one or more IBA polygons. We then calculated the IBA coverage ratio by dividing the percentage of IBA coverage within each cell or country by the total percentage of land covered by IBAs across the entire study area (i.e., 14%). The coverage ratio allowed for direct comparison with the protected area network and enabled us to identify the values of IBAs for future expansion of protected areas based on bird indicators, particularly for the potential inclusion of high‐priority areas that may not yet be formally protected but fall within recognized IBAs.

To test and compare the extent to which the current protected area network and the IBAs covered priority areas for bird conservation, we fitted three general linear models (GLMs; Appendix S14). We first fit the country‐level protected ratio and IBA coverage ratio as a function of mean priority rank per country based on population maps. Our analyses focused on proportional coverage (% of land area) instead of the absolute area (in km^2^) of protected areas or IBAs because absolute measures were strongly autocorrelated with variation in country size (Appendix S9). We then fit two similar GLMs, protected ratio and IBA coverage ratio per cell against cell priority rank. For the habitat‐specific prioritization, we tested whether the association between protected ratio and priority rank remained the same among different habitat types by fitting a GLM with cell protected ratio as a function of the interaction between habitat type and cell priority ranks based on habitat‐specific prioritization with species’ population maps (Appendix S15).

### Software environment and R packages

We conducted the spatial prioritization using Zonation v5 (Moilanen et al., 2022) and performed all data processing, spatial operations, statistical analyses, and visualization in an R environment (ver. 4.3.1). Data processing and formatting were carried out with the R packages dplyr (Wickham et al., 2023), tidyverse (Wickham et al., 2019), and reshape (Wickham, 2007). Spatial vector and raster operations were conducted using the packages sf (Pebesma, 2018), stars (Pebesma et al., 2025), and raster (Hijmans et al., 2025). Statistical modeling used the package lme4 (Bates et al., 2015), and all figures were produced using the packages ggplot2 (Wickham, 2016) and ggeffects (Lüdecke, 2018). We performed all GIS analyses under Lambert azimuthal equal‐area projection (EPSG:3035). With the exception of the EBBA2 and IBA data that are available through user agreements and the protected area data that are openly available at an EU datahub, all of the detailed data and R code for our analyses are available at GitHub (https://github.com/yanjie‐xu/zonation_birds_Europe).

## RESULTS

### Priority areas for pan‐European avian conservation

A full prioritization based on the occurrence and abundance maps of all 435 bird species showed that the important areas for bird conservation in Europe spanned a broad latitudinal range along a southwest to northeast axis from Spain to Finland. Macedonia and the Netherlands had the highest mean priority ranks, indicating the greatest potential for additional coverage of species’ ranges and populations through their protected areas (Figure 1; Appendices S1 & S2). In addition, Finland, Estonia, Spain, Albania, Bulgaria, and Latvia were also ranked among the top five countries for prioritization of new protected areas based on our separate analyses with occurrence and abundance data for breeding birds.

Protection of the top 10% of highest priority landscape based on species’ occurrence and cells with a priority rank of ≥0.9 would cover an average of 28% of species’ populations and 23% of species’ ranges across Europe (Figure 2a). Prioritization based on abundance maps would increase species’ population coverage to 34% but would slightly reduce coverage of species’ ranges to 20% (Figure 2b). If protections were extended to the top 30% of the highest priority landscape, then the coverage nearly doubled to 63% and 49% for species’ populations and ranges, respectively. According to the minimum performance curves, prioritization based on occurrence maps rather than on abundance maps was less efficient in covering both the ranges and the populations of species with limited ranges and lower abundances (Figure 2).

The three different species‐weighting schemes (SPEC, IUCN, and unweighted) emphasized different aspects of expected planning outcomes, but nevertheless, our results remained broadly consistent when applied to the same datasets (Appendix S3). On the other hand, the three different algorithms for marginal loss rules (i.e., ABF, CAZ2, and CAZMAX) affected the prioritization results. The largest difference was between the results of ABF and CAZMAX (Appendix S4), where CAZMAX gave lower priority ranks than ABF did for most areas except the edges around the study area, including regions of Italy, Norway, and Iceland.

**FIGURE 3:**
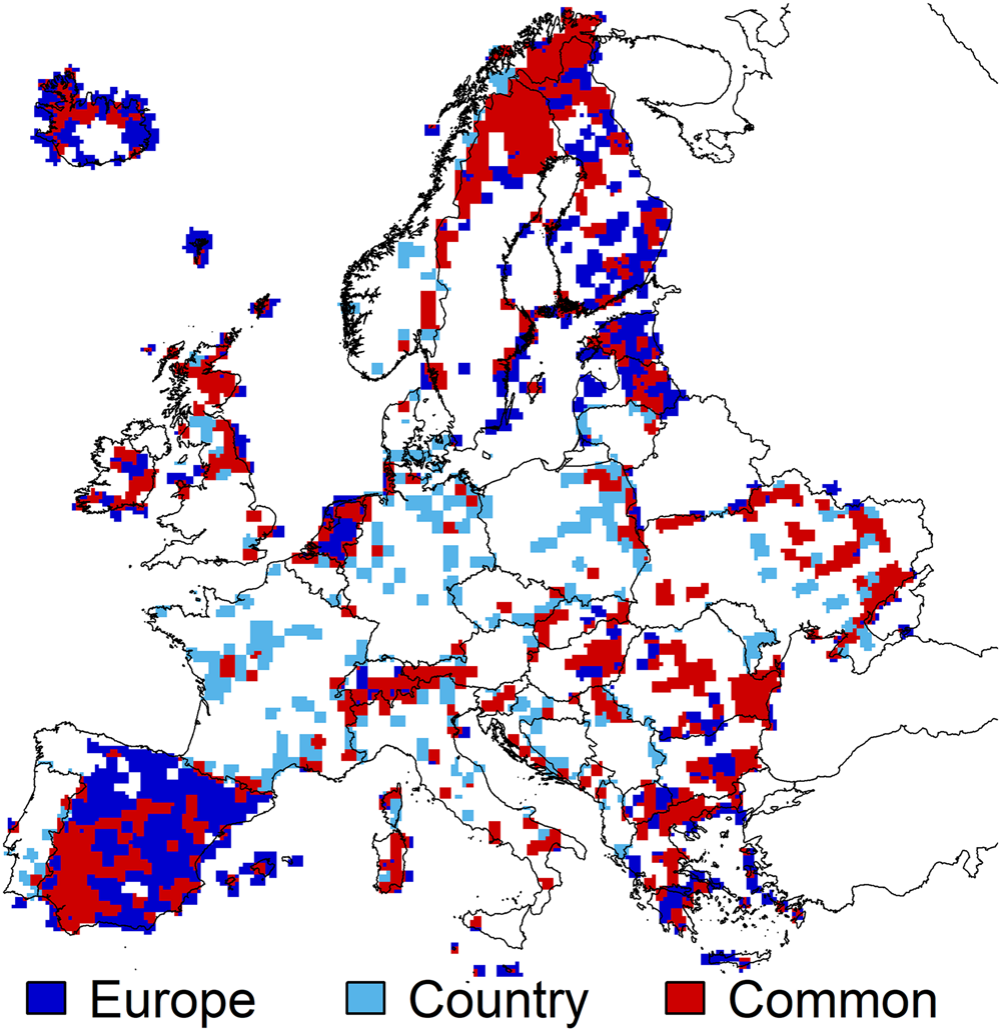
Areas representing 30% of the highest priority lands based on abundance across western Europe (dark blue), in each country (light blue), and at both spatial scales (red) (priority ranks were measured by Zonation analysis based on abundance maps of pan‐European breeding birds).

The selected priority areas clearly differed when prioritization was based on national 30% or continental 30% targets (Figure 3). The national prioritization approach resulted in more uniform coverage of priority areas than that resulting from the continental prioritization approach, which showed northern and southern sites having higher priority ranks.

### Habitat‐specific priority areas

**FIGURE 4:**
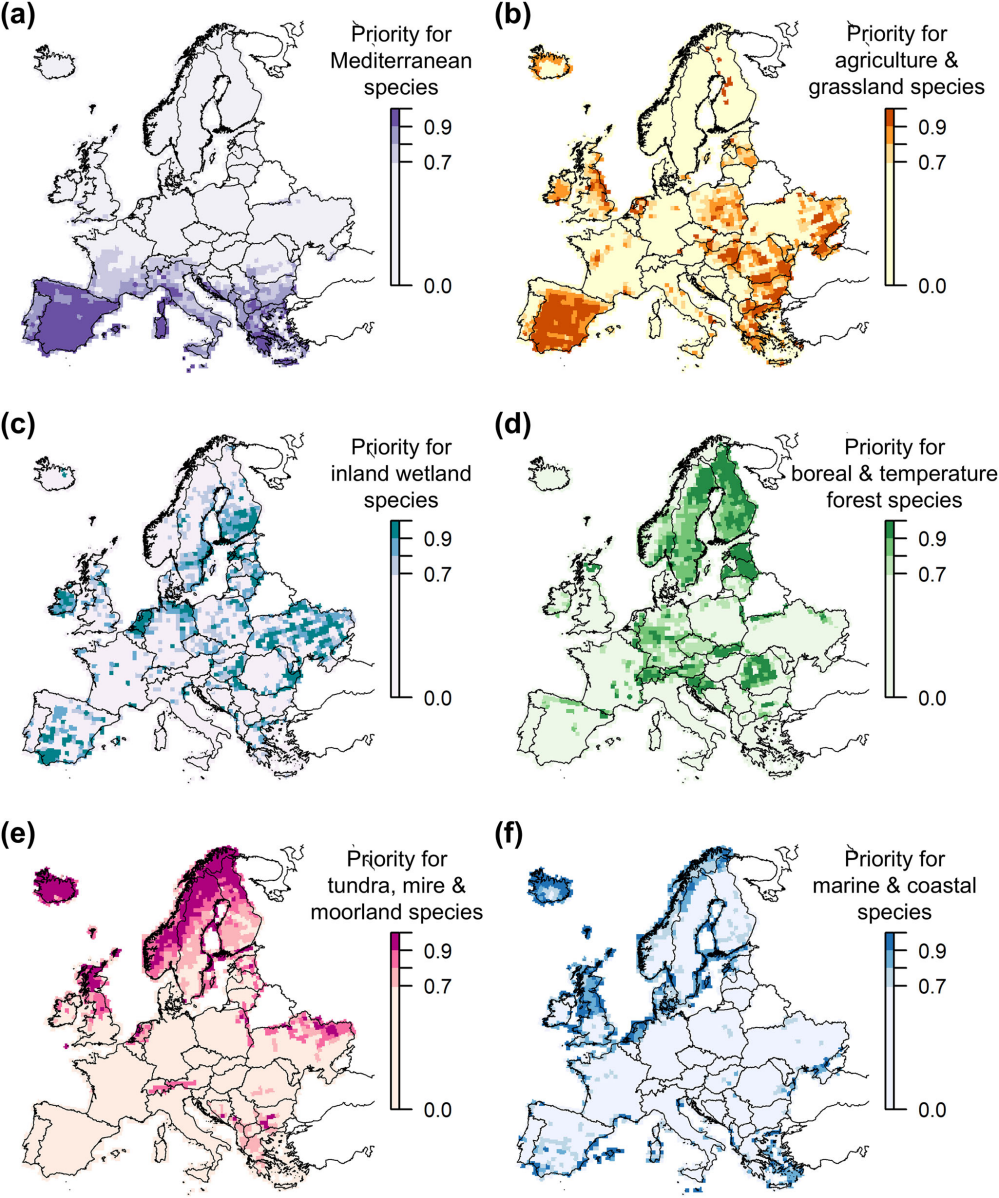
Habitat‐specific priority areas for conservation of bird populations across Europe for six different habitat types: (a) Mediterranean habitats (*N* = 39 species), (b) agriculture and grasslands (*N* = 74), (c) inland wetlands (*N* = 62), (d) boreal and temperate forests (*N* = 61), (e) tundra, mire, and moorlands (*N* = 42), and (f) marine and coastal habitats (*N* = 49).

**FIGURE 5:**
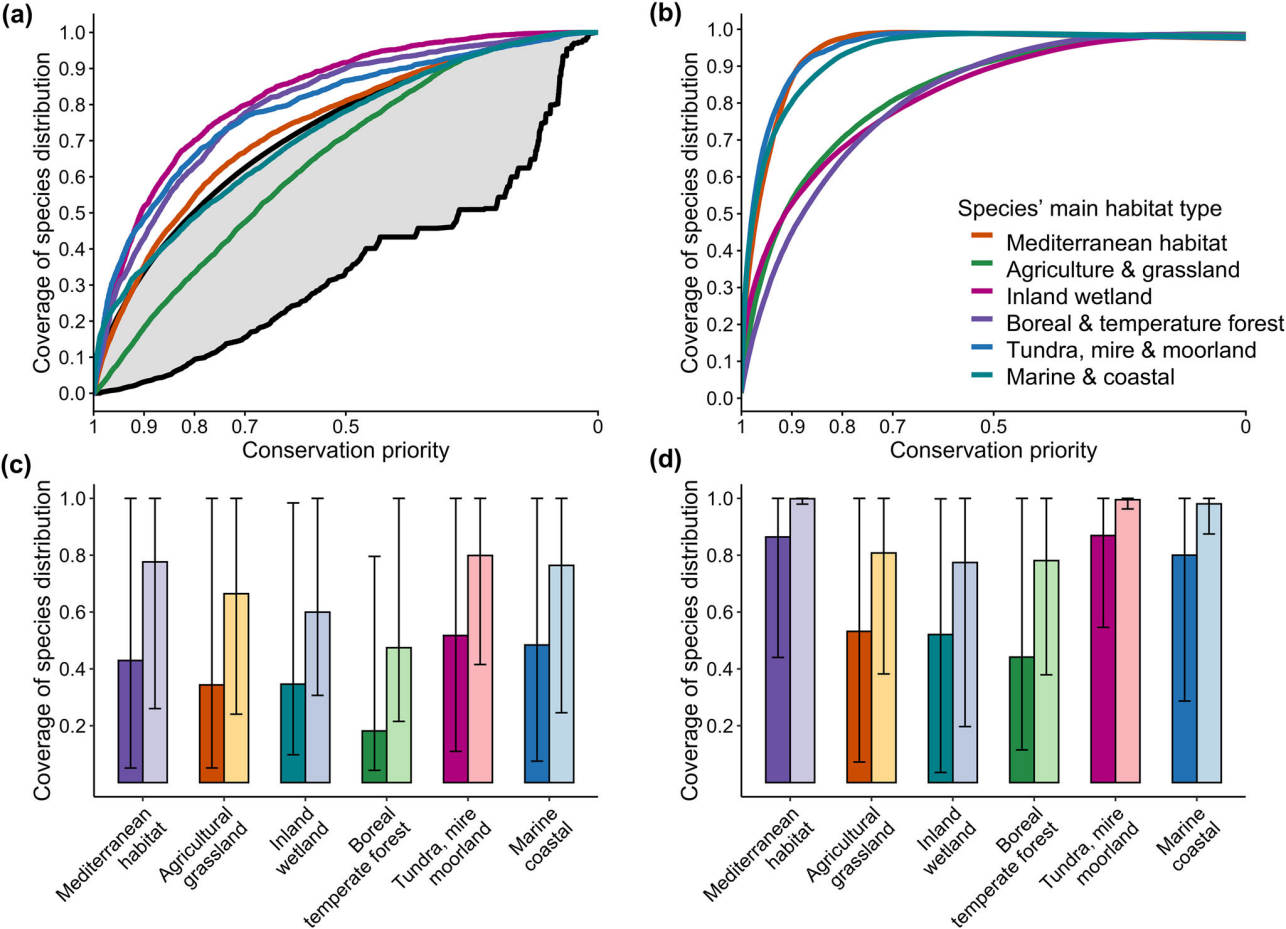
Performance curves and mean coverage for species groups with similar habitat associations based on (a) full prioritization and (b) habitat‐specific prioritization (black lines are the mean and minimum performance curve for all the species in full prioritization) and bar charts showing the mean (bars) and maximum and minimum (error bars) coverages for the species in 10% (dark‐colored) and 30% (light‐colored) of the highest priority areas based on (c) full prioritization and (d) habitat‐specific prioritization.

There were substantial differences among priority areas for the six different habitat types that followed known differences in regional distributions for major bird groups in Europe (Figure 4). Spain was the most important country for both agriculture/grassland and Mediterranean species (Appendix S6). The Netherlands, Hungary, Estonia, Latvia, and Lithuania were most important countries for inland wetland species. Five Nordic countries were important for forest, tundra, and coastal species. The coasts of mainland Norway and the Norwegian territories of Svalbard and Jan Mayen, Britain, and Ireland were important for marine and coastal birds. If conservation resources are targeted to a certain habitat type, then protecting the top 10% of the habitat‐specific priority landscapes could cover 80–87% of the species populations associated with tundra, Mediterranean, and marine habitats, whereas the overall prioritization for the complete bird community covered only 45–52% of their populations (Figure 5). Forest, grassland, and inland wetland species had lower coverage than that of species in the other habitats, with population coverages of 18%, 34%, and 35% in 10% of the highest priority areas in the full prioritization and 44%, 53%, and 52% in the habitat‐specific prioritization, respectively. When protection extended from 10% to 30% of the highest priority areas, species’ population coverage increased by 13–33%.

### Coverage of protected area network

**FIGURE 6:**
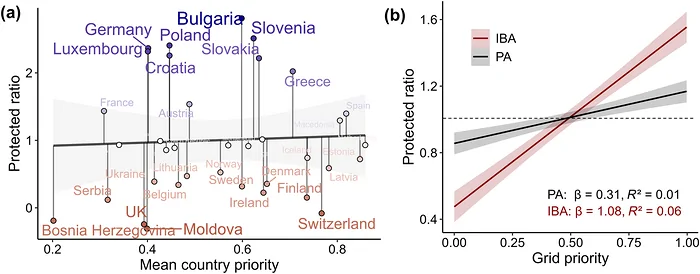
Protected area coverage of priority areas for bird populations. The fitted lines in panels (a) and (b) were the estimated relationships (a) between the mean priority rank and country‐level protected ratio and (b) between cell priority rank and protected ratio (PA, in black) or the ratio of coverage by the Important Bird and Biodiversity Areas (IBA, in red) per cell based on general linear models (GLMs). The size of the country labels was scaled by the absolute values of residuals, and their color was defined by their residuals in the GLM (negative residuals in red and positive in blue).

The current network of protected areas in the Natura 2000, Emerald Network, and nationally designated areas covers 24% of the 41 studied countries in Europe. Neither the protected ratio nor the IBA coverage ratio per country was significantly associated with its mean priority rank. The protected ratio per cell was significantly and positively associated with cell priority ranks (Figure 6; Appendix S14), which was consistent with higher rates of protection for areas with the greatest value for bird conservation. Despite a low coverage for the total study area (14%), the IBA coverage ratio had a stronger positive slope with the priority ranks and explained a larger proportion of variation in a cell's priority ranks than the protected ratio (Figure 6; Appendix S14). Many areas showed conservation gaps with the greatest shortfalls in coverage for the Nordic countries, the United Kingdom, and Ukraine (Figure 6; Appendix S10). On the other hand, priority areas were well covered by the current protected area network in Bulgaria, Germany, and Poland. As indicated by a significantly stronger positive relationship between protected ratio and cell priority ranks (Appendix S15), compared with grassland species, Mediterranean species had significantly better coverage of currently protected conservation areas. Last, tundra, wetland, and marine species were significantly less well represented than grassland species, as indicated by the negative relationships between their priority ranks and protected ratios (Appendix S11).

## DISCUSSION

Achieving the 30×30 target in Europe requires spatial prioritization that maximizes biodiversity representation while working within limited opportunities for protected area expansion (Convention on Biological Diversity, 2024; European Environment Agency, 2023). Using a unique biodiversity dataset of consistent, continent‐wide bird distribution, we developed a multi‐layered prioritization framework that contrasts occurrence‐ versus abundance‐based perspectives, evaluates trade‐offs between continental and nationally stratified 30% targets, and tests the added value of habitat‐specific prioritizations relative to a single community‐wide solution. The important areas for conservation of European birds spanned a broad geographical range and varied among habitats along an ecological gradient from Spain in southwestern continental Europe to Finland in northeastern Fennoscandia (Figures 1 & 4), reflecting the diverse climatic zones and ecosystems that support Europe's avian biodiversity and abundance. The abundance‐based prioritization increased population coverage by directing protection toward ecologically cohesive demographic strongholds, especially for range‐restricted and rare species, whereas occurrence‐based prioritization emphasized broader range edges. Our results indicated that the core population centers captured by abundance‐based prioritization were not detectable from presence‐only data alone (Johnston et al., 2015, 2020; Wolff et al., 2023). Population declines in birds and associated taxa sharing the same habitats have been widely observed (Gregory et al., 2019; Morelli et al., 2020). Compared with the full‐species prioritization for the complete bird community, the habitat‐specific prioritization identified different spatial patterns and thereby provided more efficient conservation solutions for ecosystems facing strong and distinct pressures. We highlighted gaps in the current network for regions identified as important for avian biodiversity conservation, consistent with findings from studies in other global areas (Gaston et al., 2008; Rodrigues et al., 2004). We further assessed the overlap between our priority areas and the existing network of IBAs, which are spatially delineated, biologically meaningful sites developed using standardized criteria for Europe's birds (BirdLife International, 2025a). Our abundance‐based prioritization showed that despite covering only 14% of the terrestrial area, IBAs have a stronger and more consistent spatial match with high‐priority areas than the current protected area network that covers 24% of the terrestrial area does (Figure 6b).

### Occurrence versus abundance data

Spatial prioritization based on abundance maps rather than on occurrence maps was more efficient for covering species with restricted ranges and smaller populations. Our results are consistent with those of previous studies showing that abundance‐informed prioritization covering full seasonal cycles significantly reduces the spatial extent needed to protect species effectively (Johnston et al., 2015, 2020; Wolff et al., 2023). We further determined that abundance‐informed prioritization at a continental scale can improve the representation of bird populations without increasing the area required for protection, particularly for rare and range‐restricted species (Figure 2). The results of prioritization based on occurrence versus abundance data showed a clear divergence: occurrence‐based prioritization tended to select broad regions where species are present, whereas abundance‐based prioritization concentrated protection on core population areas (Figure 1). Core population areas often coincide with ecologically stable or resource‐rich regions that support demographic viability and long‐term persistence, especially for range‐restricted or declining species (Morelli et al., 2020). However, occurrence‐based prioritization can still have merit; occurrence data are usually more available across regions and taxa, and their spatial coverage encompasses a larger portion of a species’ overall range, offering a better foundation for facilitating connectivity and dispersal among populations, which can be crucial for metapopulation viability and long‐term resilience (Brennan et al., 2022). Our findings underscore the practical importance of setting target‐based constraints in spatial prioritization tools. For example, by explicitly specifying minimum population or abundance levels for species, practitioners can better ensure that widespread yet low‐density taxa are not underrepresented in conservation planning (Carwardine et al., 2009; Kukkala & Moilanen, 2013).

### National and continental targets

Our findings showed clear spatial differences in prioritization when national targets were included, as this prioritization approach was less biased toward northern and southern areas. The difference was likely caused by the fact that at the European scale, compared with central areas, northern and southern areas have more unique species (Benedetti et al., 2022). Even though the continental‐scale prioritization may provide good overall coverage of top priority areas, we argue that national prioritization has many practical benefits. First, site selection of protected areas is typically conducted at a national level, aligning with the subsidiary principle favoring decentralized policy decisions, a core guideline in EU policy (Grupp et al., 2023). Second, national protection targets improve equality between countries as the costs of establishing new areas and benefits from improved ecosystem services and other gains are shared evenly (Bell et al., 2007; Burkhard et al., 2012). Third, more uniform coverage of protected areas across the continent can better accommodate movements of migratory species and climate change‐driven range shifts (Marjakangas et al., 2023). Even if decisions are made at a national level, international cooperation in protected area networks such as Natura 2000 and Emerald Network should be encouraged and can be effective at coordinating conservation efforts beyond national policies, thereby addressing effects of climate change and other large‐scale stressors for biodiversity loss (Gaget et al., 2022; Princé et al., 2021). Natura 2000 has effectively addressed decentralization by requiring that site designations adhere to scientific criteria, thus ensuring a science‐based approach to conservation planning (Kukkala et al., 2016). The country‐level breakdown complements recent multitaxon analyses showing that Natura 2000 effectiveness varies markedly among Member States and biogeographic regions (Hochkirch et al., 2023; Ricci et al., 2024). Both national and continental prioritizations provide concrete spatial guidance on where additional national or transboundary measures would yield the largest gains for bird populations, especially if habitat‐specific conservation needs and connectivity between protected sites are considered.

### Benefit of habitat‐specific prioritization

Significant variation in the distribution of priority areas across different habitats (Figure 4) highlights a need for habitat‐specific prioritization to effectively tailor conservation efforts to the unique requirements and threats of each habitat (Sutherland et al., 2009). Tundra and marine ecosystems exhibited lower coverage in protected areas than would be expected for an optimal distribution of conservation areas for biodiversity, especially compared with coverage of agriculture and grassland, forest, and Mediterranean habitats in protected areas (Appendix S11). The conservation gaps can be efficiently addressed using our habitat‐specific prioritization maps, as the top 10% highest priority landscapes identified are sufficient to cover most breeding populations of tundra and marine species (Figure 5d). Recent conservation efforts appear less effective for farmland birds, which are declining rapidly across Europe, than for species associated with other habitats (Brlík et al., 2021). Both farmland and forest birds have showed significant population declines between 1990 and 2023 across Europe (Common Bird Index in Europe). These two groups of birds have broad, diffuse distributions across climatic zones that make them less compatible with cost‐efficient area selection. Indeed, our results suggest that farmland and forest birds were covered less than the species associated with the other habitats in the top 30% of the highest priority areas (Figure 5), which was consistent with the results of studies highlighting the difficulty of capturing widespread, low‐density, and habitat‐generalist species through protected area networks alone (Benedetti et al., 2022; Morelli et al., 2020). Our finding highlights the need for conservation efforts to go beyond the 30×30 protected area target to ensure effective coverage of widespread species through additional habitat management and restoration, and other effective area‐based conservation measures (OECMs) (Gurney et al., 2021). For farmland species, conservation depends more on landscape practices than on protected areas (Pe'er et al., 2019), and OECMs offer an efficient tool for integrating biodiversity objectives with socioeconomic and cultural values by aligning conservation with local livelihoods and service provision (Gurney et al., 2021).

Our prioritization results have at least three sources of uncertainty. Some of the differences in rankings between grid cells and countries in our prioritization maps may reflect differences in data collection efforts, some of which could be integrated into future modeling. Although variation in sampling effort may affect comparisons at a local level and underscores the need for careful interpretation of results, this variation did not impact the broader‐scale continental patterns found in this study. Models of species distributions can mediate the effect of spatial sampling biases, but their potential discrepancy with actual species distributions can lead to ineffective spatial prioritization plans (Soultan et al., 2022). Here, we focused specifically on the coverage of species distributions and abundances, recognizing that spatial extent represents only one dimension of biodiversity and may not fully capture other important environmental values, such as features relevant to ecological integrity, ecosystem processes, or connectivity. Furthermore, the robustness of results from conservation prioritization based on static occurrence and abundance maps may be reduced by climate or land‐use changes that drive shifts in species distributions (Howard et al., 2023; Marjakangas et al., 2023). Extending our continental‐scale prioritization framework over time would clarify how priorities shift under climate change, a critical need given that European protected areas are projected to face rapidly intensifying pressures that may reduce their long‐term effectiveness without adaptation (Cimatti et al., 2025).

Our prioritization maps and framework offer a valuable foundation for proceeding with finer‐scale conservation area planning, restoration, and habitat management for species associated with habitats under anthropogenic pressures (Langhammer et al., 2024). Conducting a spatial assessment at a 50 × 50‐km resolution is appropriate for standardized, continent‐wide analysis, but most existing protected areas are smaller than this grid cell size (Schauman et al., 2024), making direct implementation challenging without additional refinements. Nevertheless, the strong spatial correspondence between IBAs and high‐priority areas suggests that IBAs represent efficient candidates for strengthened protection status or recognition through OECMs, particularly in the United Kingdom, Norway, and Switzerland, where shortfalls are most pronounced. Our analysis focused on breeding birds, but that should not limit the broader relevance of our findings, as birds are central to European biodiversity policy and are widely recognized as reliable indicators of wider biodiversity patterns (BirdLife International, 2025b; Fraixedas et al., 2020). Future work should build on this framework by integrating finer‐resolution national biodiversity data with spatially explicit information on habitat condition and management constraints, enabling continental priorities to be refined into implementable area‐based conservation targets. Extending the framework to additional taxa and repeating prioritization under projected land use and climate change would further clarify where conservation networks are likely to remain effective and where adaptive responses are most urgently needed.

## Supporting information




**Supplementary Materials**.
