# Supplementary material for: Continental‐wide prioritization of protected areas to address global and habitat‐specific goals for avian biodiversity conservation
Source: Conserv Biol. 2026 May 14;40(5):e70324. doi: 10.1111/cobi.70324 (PMC13613562; doi:10.1111/cobi.70324)
Supplement: Supplementary file 1 [file COBI-40-e70324-s001.docx]

Supporting information for:

Continental-wide prioritization of protected areas to approach global and habitat-specific goals for avian biodiversity conservation

**Appendix S1.** Priority countries for the ranges of European breeding birds. The bars are the mean priority ranks of each country based on species’ occurrence maps, with 1 ranked as the most valuable and 0 as the least valuable.

**Appendix S2.** Priority countries for the population of European breeding birds. The bars are the mean priority ranks of each country based on species’ abundance maps, with 1 ranked as the most valuable and 0 as the least valuable.

**Appendix S3.** Sensitivity analysis for species weights in Zonation prioritization with Additive Benefit Function (ABF) based on species’ abundance maps. (a) The prioritization result without species’ weights; (b) The difference between priority ranks based on Zonation with SPEC category as species’ weights and without weights; (c) The difference between priority ranks based on Zonation with IUCN Europe category as species’ weights and without weights.

**Appendix S4.** Sensitivity analysis for different marginal loss rules in Zonation algorithm without species’ weights based on species range maps. (a) ABF – Additive Benefit Function sums weighted marginal losses across all species per cell, removing cells with lowest losses first. The map shows the prioritization result from ABF; (b) CAZ2– Core-Area Zonation variant 2 bases cell removal on the maximum marginal loss among species in that cell but uses a variant aggregation method designed to balance representation (retaining both average and minimum coverage). The map shows the difference between results from CAZ2 and ABF; (c) CAZMAX uses the standard core-area rule, removing cells based on the single most sensitive species, thereby prioritizing core habitats of species with smallest ranges. The map shows the difference between results from CAZ2 and ABF.

**Appendix S5.** Habitat-specific priority areas for conservation of bird ranges across Europe. The priority maps across the study area were estimated by species of six different habitat types: (a) Mediterranean habitats, (b) agriculture and grasslands, (c) inland wetlands, (d) boreal and temperate forests, (e) tundra, mire, and moorlands, (f) marine and coastal habitats.

**Appendix S6.** Habitat-specific priority countries for conservation of bird ranges across Europe. The mean priority ranks per country were estimated by species of six different habitat types: (a) Mediterranean habitats, (b) agriculture and grasslands, (c) inland wetlands, (d) boreal and temperate forests, (e) tundra, mire, and moorlands, (f) marine and coastal habitats.

**Appendix S7.** Habitat-specific priority countries for conservation of bird abundance across Europe. The mean priority ranks per country were estimated by species of six different habitat types: (a) Mediterranean habitats, (b) agriculture and grasslands, (c) inland wetlands, (d) boreal and temperate forests, (e) tundra, mire, and moorlands, (f) marine and coastal habitats.

**Appendix S8.** The relationship between mean country priority values based on abundance Zonation and the country’s coverage ratio by the Important Bird and Biodiversity Areas (IBA). The fitted line (GLM) were the estimated associations between the mean priority and IBA coverage over a country. The shaded area is the 95% confidence interval of the GLM estimate. The scatter points are the original data points of each country, their colour was defined by their residuals in the corresponding GLM (negative residuals in red and positive in blue), and the size of the country labels were scaled by the absolute values of residuals. The statistical details from the fitted GLM are listed in Table S4.

**Appendix S9.** Total protected area depends on total area of a country. The fitted line (GLM) were the estimated associations between the total area and protected area over a country. The shaded area is the 95% confidence interval of the GLM estimate. The scatter points are the original data points of each country, their colour was defined by their residuals in the corresponding GLM (negative residuals in red and positive in blue), and the size of the country labels were scaled by the absolute values of residuals. The statistical details from the fitted GLM are listed in Table S3.

**Appendix S10.** The map shows the protected ratio (LowProt: protected ratio < 1; HighProt: protected ratio ≥ 1) versus priority rank per cell (classified as top 10%, top 30%, and others).

**Appendix S11.** The fitted lines were the estimated relationships between the habitat-specific priority rank and protected ratio at a cell level. Positive slopes mean that as the priority rank of a cell increases, the proportion of the cell that is protected also increases. The strength and direction of this relationship can differ depending on habitat type, since the model includes interactions. The shaded areas are the 95% confidence interval of the GLM estimates.

**Appendix S12.** Species weights based on Species of European Conservation Concern (SPEC) categories defined by assessments on their global extinction risk and European proportion (Burfield et al. 2023).

| SPEC category | Abbreviation | Weight |
| --- | --- | --- |
| Species of global concern | SPEC1 | 4 |
| Species of European concern and concentrated in Europe | SPEC2 | 3 |
| Species of European concern but not concentrated in Europe | SPEC3 | 2 |
| Species not categorised as SPEC | Non-SPEC | 1 |

**Appendix S13.** Species weights based on species’ IUCN Europe category defined in European red list of birds by European Commission and Directorate-General for Environment (BirdLife International 2021)

| IUCN category | Abbreviation | Weight |
| --- | --- | --- |
| Critically Endangered | CR | 5 |
| Endangered | EN | 4 |
| Vulnerable | VU | 3 |
| Near Threatened | NT | 2 |
| Least Concern | LC | 1 |
| Regionally Extinct | RE | Excluded |

**References for Table S1 and S2**

1. Burfield, I. J., C. A. Rutherford, E. Fernando, H. Grice, A. Piggott, R. W. Martin, M. Balman, M. I. Evans, and A. Staneva (2023). Birds in Europe 4: the fourth assessment of Species of European Conservation Concern. Bird Conservation International 33:e66.
2. BirdLife International (2021). European Red List of Birds. Luxembourg: Publications Office of the European Union. Available at: <https://www.birdlife.org/wp-content/uploads/2022/05/BirdLife-European-Red-List-of-Birds-2021.pdf.pdf>

**Appendix S14.** Statistical estimates for the regressions of priority ranks for conservation of bird populations (based on species’ abundance maps) as a function of protected ratio and coverage by the Important Bird and Biodiversity Areas (IBAs) of each country and each cell (Figure 6a).

| Model | Estimate | SE | t-value | P-value |
| --- | --- | --- | --- | --- |
| Protected ratio ~ mean priority ranks per country  N = 36, R^2^ = 0.002 | | | | |
| Intercept | 0.937 | 0.253 | 3.706 | <0.001* |
| Mean priority | 0.116 | 0.435 | 0.222 | 0.791 |
| IBA coverage ratio ~ mean priority ranks per country  N = 36, R^2^ = 0.005 | | | | |
| Intercept | 0.633 | 0.426 | 1.485 | 0.147 |
| Mean priority | 0.991 | 0.733 | 1.351 | 0.186 |
| Protected ratio ~ mean priority ranks per grid  N = 2826, R^2^ = 0.010 | | | | |
| Intercept | 0.856 | 0.033 | 25.548 | <0.001* |
| Grid priority | 0.314 | 0.058 | 5.363 | <0.001* |
| IBA coverage ratio ~ mean priority ranks per grid  N = 2826, R^2^ = 0.058 | | | | |
| Intercept | 0.475 | 0.047 | 10.120 | <0.001* |
| Grid priority | 1.082 | 0.082 | 13.210 | <0.001* |
| Total protected area per country ~ total country area  N = 36, R^2^ = 0.785 | | | | |
| Intercept | 422.8 | 4690.0 | 0.109 | 0.914 |
| Country area | 0.229 | 0.021 | 12.129 | <0.001* |

**Appendix S15.** Statistical estimates for the regression coefficients of interaction of habitat type and priority ranks based on habitat-specific prioritizations on protected ratio per grid (Figure 6bd).

| Model | Estimate | SE | t-value | P-value |
| --- | --- | --- | --- | --- |
| Protected ratio ~ habitat × priority ranks per grid  N = 16956, R^2^ = 0.012, reference level: agriculture and grassland | | | | |
| Intercept | 0.868 | 0.034 | 25.785 | <0.001* |
| Grid priority | 0.286 | 0.058 | 4.883 | <0.001* |
| Boreal & temperate forest | 0.015 | 0.048 | 0.320 | 0.749 |
| Tundra, mire & moorland | 0.313 | 0.048 | 6.560 | <0.001* |
| Mediterranean | -0.166 | 0.048 | -3.474 | <0.001* |
| Marine & coastal | 0.209 | 0.048 | 4.383 | <0.001* |
| Inland wetland | 0.241 | 0.048 | 5.076 | <0.001* |
| Grid priority × Boreal & temperate forest | -0.030 | 0.083 | -0.364 | 0.716 |
| Grid priority × Tundra, mire & moorland | -0.628 | 0.083 | -7.578 | <0.001* |
| Grid priority × Mediterranean | 0.328 | 0.083 | 3.967 | <0.001* |
| Grid priority × Marine & coastal | -0.420 | 0.083 | -5.054 | <0.001* |
| Grid priority × Inland wetland | -0.485 | 0.083 | -5.866 | <0.001* |
